# Identification and Characterization of Microsatellite Markers Derived from the Whole Genome Analysis of *Taenia solium*

**DOI:** 10.1371/journal.pntd.0004316

**Published:** 2015-12-23

**Authors:** Mónica J. Pajuelo, María Eguiluz, Eric Dahlstrom, David Requena, Frank Guzmán, Manuel Ramirez, Patricia Sheen, Michael Frace, Scott Sammons, Vitaliano Cama, Sarah Anzick, Dan Bruno, Siddhartha Mahanty, Patricia Wilkins, Theodore Nash, Armando Gonzalez, Héctor H. García, Robert H. Gilman, Steve Porcella, Mirko Zimic, Cysticercosis Working Group in Peru

**Affiliations:** 1 Laboratorio de Bioinformatica y Biologia Molecular, Laboratorios de Investigación y Desarrollo, Facultad de Ciencias y Filosofía, Universidad Peruana Cayetano Heredia, Lima, Peru; 2 Genomics Unit, Research Technologies Section, Rocky Mountain Laboratories, NIAID, NIH, Hamilton, Montana, United States of America; 3 Biotechnology Core Facility Branch, National Center for Emerging and Zoonotic Infectious Diseases, Centers for Disease Control and Prevention, Atlanta, Georgia, United States of America; 4 Division of Parasitic Diseases and Malaria, Center for Global Health, Centers for Disease Control and Prevention, Atlanta, Georgia, United States of America; 5 Facultad de Medicina Veterinaria, Universidad Nacional Mayor de San Marcos, Lima, Peru; 6 Departamento de Microbiología, Facultad de Ciencias y Filosofía, Universidad Peruana Cayetano Heredia, Lima Peru; 7 Instituto Nacional de Ciencias Neurológicas. Lima, Peru; 8 Department of International Health, Bloomberg School of Public Health, Johns Hopkins University, Baltimore, Maryland, United States of America; University of Würzburg, GERMANY

## Abstract

**Background:**

Infections with *Taenia solium* are the most common cause of adult acquired seizures worldwide, and are the leading cause of epilepsy in developing countries. A better understanding of the genetic diversity of *T*. *solium* will improve parasite diagnostics and transmission pathways in endemic areas thereby facilitating the design of future control measures and interventions. Microsatellite markers are useful genome features, which enable strain typing and identification in complex pathogen genomes. Here we describe microsatellite identification and characterization in *T*. *solium*, providing information that will assist in global efforts to control this important pathogen.

**Methods:**

For genome sequencing, *T*. *solium* cysts and proglottids were collected from Huancayo and Puno in Peru, respectively. Using next generation sequencing (NGS) and *de novo* assembly, we assembled two draft genomes and one hybrid genome. Microsatellite sequences were identified and 36 of them were selected for further analysis. Twenty *T*. *solium* isolates were collected from Tumbes in the northern region, and twenty from Puno in the southern region of Peru. The size-polymorphism of the selected microsatellites was determined with multi-capillary electrophoresis. We analyzed the association between microsatellite polymorphism and the geographic origin of the samples.

**Results:**

The predicted size of the hybrid (proglottid genome combined with cyst genome) *T*. *solium* genome was 111 MB with a GC content of 42.54%. A total of 7,979 contigs (>1,000 nt) were obtained. We identified 9,129 microsatellites in the Puno-proglottid genome and 9,936 in the Huancayo-cyst genome, with 5 or more repeats, ranging from mono- to hexa-nucleotide. Seven microsatellites were polymorphic and 29 were monomorphic within the analyzed isolates. *T*. *solium* tapeworms were classified into two genetic groups that correlated with the North/South geographic origin of the parasites.

**Conclusions/Significance:**

The availability of draft genomes for *T*. *solium* represents a significant step towards the understanding the biology of the parasite. We report here a set of *T*. *solium* polymorphic microsatellite markers that appear promising for genetic epidemiology studies.

## Author Summary

*Taenia solium*, the pork tapeworm, is an important pathogen as it is a major cause of acquired epilepsy in developing countries. The parasite was eliminated from most developed countries decades ago due to improvement in sanitary conditions but it remains a common infection across Asia, Africa and Latin America. Identification of genetic variants within *T*. *solium* will enable to study the genetic epidemiology, distribution and movement of this parasite within endemic communities, which will ultimately facilitate the design of control strategies to reduce the health and economic burden of disease.

Microsatellites have been used in other parasites to identify genetic variants. In this study, we partially sequenced the genome of *T*. *solium* and identified microsatellites widely distributed in the genome using bioinformatics tools. We evaluated the distribution of these microsatellites collected from 20 tapeworms from the north and 20 tapeworms from the south of Peru. We identified seven polymorphic microsatellites, and evaluated their capacity to differentiate genetic variants of *T*. *solium*. Interestingly, tapeworms from the North and South of Peru showed different genotypes, suggesting its use as a potential marker to differentiate geographic origin.

## Introduction

Cysticercosis is an infection caused by the larval stage of the cestode *Taenia solium*. When the larval stages infect the central nervous system, the infection is known as neurocysticercosis (NCC) and is the most common cause of adult-onset seizures in endemic regions worldwide. Crude estimates of the burden of infection and disease suggest that greater than ten million people have NCC and as many as 2.7–5.6 million suffer from epilepsy [1]. A recent analysis concluded that in Latin America, vast parts of Asia, the Indian subcontinent and Southern China, Sub-Saharan Africa, and Oceania, 29% of all cases of epilepsy are attributable to NCC [2].

Humans are the only known definitive host, harboring the adult tapeworm and releasing infectious eggs to the environment [3]. In pigs that ingest infectious ova or proglottids, the released oncospheres cross the intestinal wall into the circulatory system where they become trapped in the microcapilaries, often in the brain, muscles and subcutaneous tissues. The oncospheres develop into cysticerci (cysts) and if present in the parenchyma of the brain, seizures and epilepsy may occur as a result of host inflammation against the cysts.

Understanding the genetic variation of *T*. *solium* has the potential to improve our knowledge of the biology, epidemiology, infectivity, and pathogenicity of this parasite in endemic regions [4–7]. Moreover, analysis of the genetic variation within and between different geographical populations can provide information on evolution [8], genetic differentiation and speciation of parasites [9], as well as provide tools for understanding transmission dynamics, which may contribute to public health efforts to control this parasitic infection.

The first attempts at genotyping *Taenia* parasites were directed towards the differentiation of *Taenia* species based on the sequence polymorphism of mitochondrial NADH dehydrogenase 1 and cytochrome c oxidase subunit I (COI) genes using single-strand conformation polymorphism (SSCP) [10]. Restriction fragment length polymorphism (RFLP) also was used to discriminate *Taenia* species by analyzing the ribosomal 5.8S gene sequence as well as the internal transcribed spacer (ITS) [11]. In 2001, Hancock *et al* showed diversity among *T*. *solium* cysts from different countries using COI, a portion of the ITS1 encoded gene and the diagnostic antigen Ts14. Little genetic diversity within *T*. *solium* samples collected from South America and Asia was observed. In addition, 15 isolates from Peru had similar COI sequences showing no genetic variability between them [12]. Later, two different worldwide genotypes were reported, with Asian parasites grouped into one cluster, and parasites from Latin America and Africa grouped into another cluster [4]. It has been suggested that the low variation found in *T*. *solium* isolates may be associated with the limited resolution of the experimental techniques used at that time [8].

More recently, with the development of new DNA analysis tools such as Random Amplification of Polymorphic DNA (RAPD), greater genetic variation has been reported in parasites from communities in Mexico, Honduras and Madagascar [5,13–15]. This data suggests that *T*. *solium* has local lineages with different genetic characteristics. However, some disadvantages have been reported with RAPD such as low reproducibility, inability to test heterozygosity and subjective interpretation of the data [16,17]. Therefore, a more robust tool with higher resolution is needed to obtain more precise genotyping of *T*. *solium* isolates.

Microsatellites, or Simple Sequence Repeats (SSR), are repetitive DNA sequences consisting of blocks of 1 to 6 nucleotides repeated up to 60 times 8. They are highly polymorphic in the number of repeated units. The variation in size of repeat domains is mainly generated by slippage of DNA polymerase during DNA replication, resulting in the insertion or deletion of one or more repeated units [18,19]. Microsatellites have the advantage of being multi-loci and principally neutral markers [19], meaning that unlike protein-encoding genes, they are less likely to be subject to selective pressure. Microsatellites are highly reproducible and specific, and are easily identified from genome sequences by bioinformatics data mining [20–22].

Microsatellite polymorphisms can be detected by polymerase chain reaction (PCR) amplification followed by DNA electrophoresis [8,23]. This technique has been used to analyze genetic variation of other parasites such as *Leishmania* spp. [24], *Schistosoma japonicum* [6], *Trypanosoma cruzi* [25], and *Plasmodium falciparum* [26]. Microsatellite markers also have the advantage of being able to detect greater genetic variation than other genetic markers, as has been demonstrated in *Echinococcus multilocularis* [27]. This work suggests that microsatellite polymorphism analysis is an appropriate tool to differentiate *T*. *solium* isolates. With the availability of draft genome sequences, the identification of microsatellites is more efficient [20].

Recently, a draft genome of a *T*. *solium* isolate recovered from Mexico has been published [28]. It is however necessary to have more genomic information available, in order to identify genotyping markers.

In this study we present two draft genome sequences corresponding to *T*. *solium* specimens from Huancayo (cysts) and Puno (proglottid) from which we identified and characterized microsatellite markers. We explore microsatellite length variability to differentiate *T*. *solium* isolates from two regions of Peru. To analyze the microsatellites length we used a multi-capillary electrophoresis QIAxcel system that has the advantage of automatized size determination [29]. Although its advantages, this technique is limited by 3–5bp resolution that will not let us differentiate length polymorphism lower than 3–5 bp. The proposed microsatellites will lead to a more comprehensive understanding of the epidemiology of this important human pathogen.

## Materials and Methods

### *Taenia solium* tissue specimens for genome sequencing

**Fig 1:**
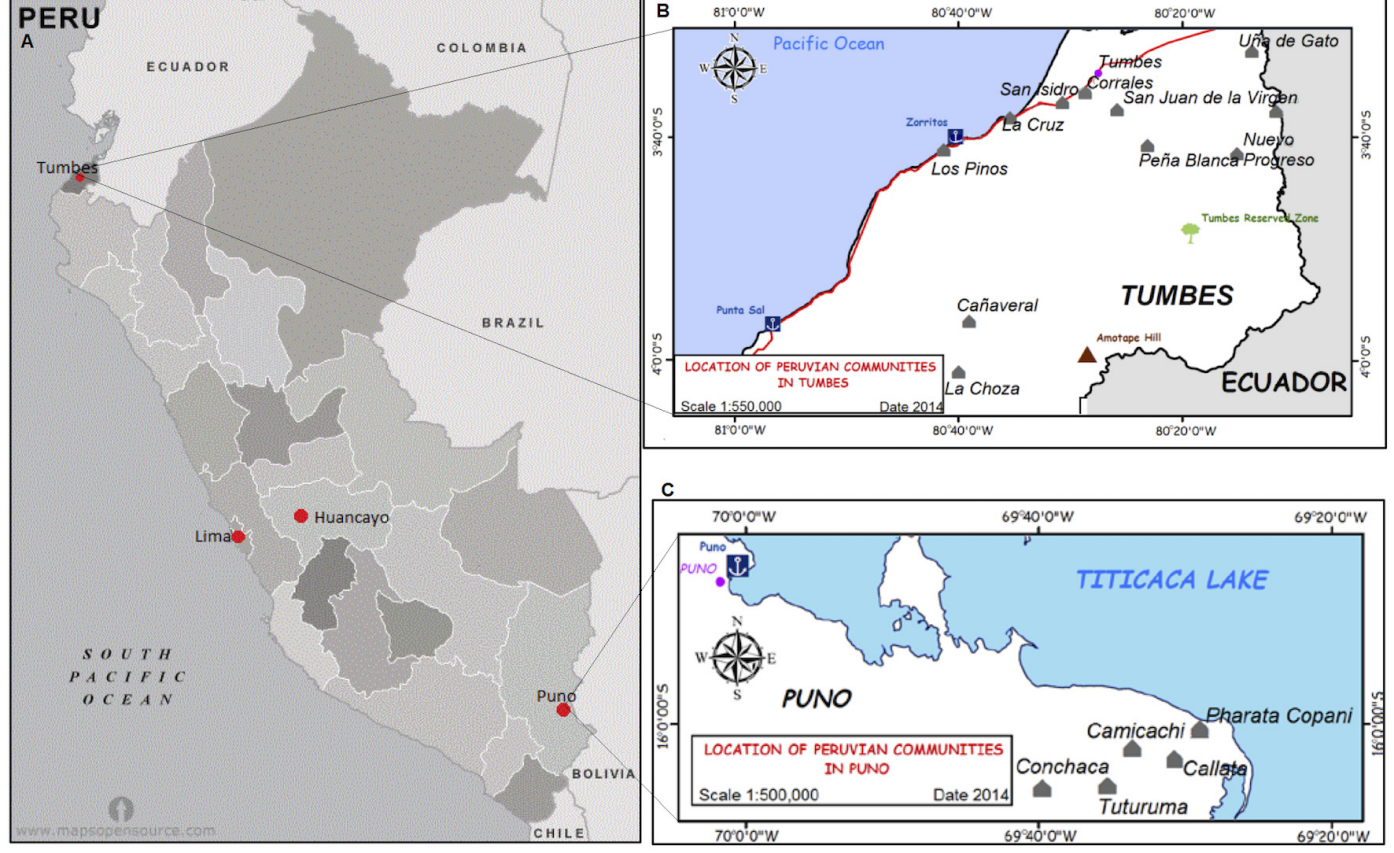
Map of Peru depicting the location of *T*. *solium* isolates used for this study. Map showing the location of Tumbes and Puno, as well as Huancayo (locality of one of the genome source) and Lima, the country´s capital (A). Maps of Tumbes (B) and Puno (C) showing the location of the communities of origin of the tapeworms for this study

Individual cysticerci (cysts) were recovered from a single, naturally infected pig from Huancayo, a city in the central Andean region. One proglottid from Puno (Fig 1) was excised from segments of an adult tapeworm recovered from a single fecal specimen and used for extraction of DNA. To minimize contamination with exogenous materials, the proglottid was washed thoroughly 10 times with phosphate buffer solution (PBS), transported in a mixture of PBS and antibiotics penicillin/streptomycin/ amphotericin B), and stored at -70°C until the DNA was extracted.

### Extraction of genomic DNAs from Huancayo cysts and Puno proglottids

#### DNA extraction from the Huancayo cyst sample

DNA and RNA was extracted simultaneously from *T*. *solium* Huancayo cysts (named “Huancayo-cyst”) at Rocky Mountain Laboratories (RML) (NIH, NIAID) following the Qiagen AllPrep DNA/RNA Mini kit (Qiagen, Valencia, CA) protocol with the following modifications. Cysts were first frozen in liquid nitrogen and finely ground to a powdery-like texture using a liquid nitrogen chilled mortar and pestle. The ground tissue was transferred to a 1.5 mL Eppendorf tube containing 1.0 mL RLT buffer (cell lysis buffer for DNA/RNA extraction, Qiagen) supplemented with 0.143 M β-mercaptoethanol and allowed to incubate at room temperature for 15 minutes with occasional gentle vortexing. Following the incubation, the extract was homogenized using a QIAshredder spin column (Qiagen) and then purified with AllPrep DNA/RNA kit reagents following the manufacturer’s protocol. DNA and RNAs were both eluted twice with 50 μl elution buffer per elution. DNA yield and purity was assessed using dsDNA quantitation (Life Technologies, Grand Island, NY) and UV spectrophotometry at A260 nm and A280, respectively. DNA quality was visualized on a 1% agarose gel (Lonza, Rockland, ME). Portions of the DNA samples from the cysts were all pooled into one tube in order to meet quantity and quality thresholds required for downstream Next Generation Illumina sequencing. RNAs were stored at -80°C for future mRNA analyses.

#### DNA extraction from the Puno proglottid sample

DNA was extracted from a *T*. *solium* proglottid originating from Puno (named “Puno-proglottid”) at the Centers for Disease Control and Prevention (CDC). Genomic DNA was extracted using a phenol chloroform extraction method, which included a lysozyme treatment at 37°C for 1 hour to minimize/eliminate bacterial contamination of proglottids, since those were recovered from human feces, followed by incubation with Proteinase K at 56°C overnight. The quantity and quality of the eluted DNA was initially tested by ultraviolet-Vis spectrophotometry (Nanodrop, Thermo Scientific, Newark, DE), and further evaluated with a picogreen kit (Life Technologies, Cat N°P7598) and Biophotometer Plus (Eppendorf), respectively. In addition, for quality analysis, one microliter of the eluted DNA was evaluated by electrophoresis through a 2% agarose gel.

### Next Generation library construction and sequencing procedure

#### Sequencing of the Huancayo-cysts

For the Huancayo-cyst DNA, a paired-end and mate-pair library were constructed separately and run on an Illumina HiSeq 2000 (Illumina Inc, San Diego CA) at RML/NIH. The paired-end library was prepared as follows; one microgram of pooled Huancayo cyst DNA was processed using the TruSeq DNA Sample Preparation Guide, Rev. A., November 2010 (Illumina Inc., San Diego, CA). The resulting library was clustered on a cBot using a paired-end flowcell and sequenced 100 cycles (bases) from both ends. The mate-pair library was prepared and sequenced in the following manner; approximately 4 micrograms of pooled Huancayo-cyst DNA was sheared using a Hydroshear device (Digilab Inc., Marlborough, MA) and processed following the procedure provided in Preparing 2–5kb Samples for Mate Pair Library Sequencing, Rev. B, February 2009 (Illumina Inc., San Diego, CA). The biotin-labeled fragments were electrophoresed on a 0.8% TAE preparatory gel and fragments ranging in size from 2.4 to 4 Kb were excised for the circularization reaction. The resulting library was clustered on a cBot using a paired-end flowcell and sequenced 100 cycles (bases) from both ends.

#### Sequencing of the Puno-proglottid

For the Puno-proglottid DNA a fragment sequencing approach using the 454 GS-FLX+ (Roche Applied Science, Indianapolis, IN) and Illumina Genome Analyzer IIe (Illumina Inc., San Diego, CA) was employed at the CDC. The 454-FLX Titanium shotgun library was prepared as follows; DNA fragments larger than 1.5 Kb were extracted from the Puno proglottid genomic DNA and 500 ng was processed using the procedure in the Rapid Library Preparation Method Manual, GS FLX+ Titanium Series, October 2009 (454 Life Sciences, Branford, CT). For emulsion PCR a 4 copy-per-bead ratio was used to yield a bead enrichment of 8%. The resulting enriched beads were then sequenced using 454 GS FLX Titanium chemistry. The Illumina fragment library was prepared and sequenced in the following manner: one microgram of Puno proglottid genomic DNA was processed using TruSeq DNA Sample Preparation Guide, Rev. A., November 2010 (Illumina Inc., San Diego, CA). The resulting library was clustered on a cBot using a single-read flowcell and sequenced 100 cycles (bases).

### Huancayo-cyst genome assembly

For the assembly, both the Illumina mate-pair and paired-end sequencing results were combined, providing a total of 878,340,445 usable reads at 100 bp average length. In order to provide the best assembly at an average of 157X coverage and for an expected genome size of 115MB, a subset of 175,931,369 reads were selected for de novo assembly process using Velvet v1.1.05 [30], with the following parameters: coverage cutoff = 10, expected coverage = 26, paired end insert length = 350, and mate paired insert length = 3,500.

### Puno-proglottid genome assembly

For the Puno proglottid genome assembly, both the Illumina mate-pair and the 454 GS FLX raw signals data were processed with the software GS Run Processor to obtain the reads, which were assembled using GS de novo Assembler 2.6 with the following parameters: minimum read length = 20 nucleotides (nt), overlap seed step = 12 nt, overlap seed length = 16 nt, overlap minimum match length = 40 nt, overlap minimum match identity = 90 nt, overlap match identity score = 2 nt, overlap match difference score = -3 nt, all contig threshold = 100 nt, large contig threshold = 500 nt; with an expected depth of 25X. Duplicated reads were used and the assembly was performed 30 times, taking the iteration with the mean number of contigs.

### Cysticercus/proglottid hybrid genome assembly

To generate a more complete *T*. *solium* genome sequence, we produced a hybrid assembly consisting of the Puno-proglottid fragment 454 reads combined with the Huancayo-cyst Illumina paired-end reads as follows. The hybrid genome was generated using Velvet v1.1.05 de novo assembler using the Puno-proglottid 454 fragment reads, Huancayo-cyst Illumina paired-end reads, and Huancayo-cyst Illumina mate-pair reads. Velvet parameters used are as follows: kmer value = 57, coverage cut-off = 9, expected coverage = 38, paired insert length = 350, and mate pair insert length = 3500.

### Public submission of genomes assemblies

The set of contigs corresponding to the genome of the Huancayo-cysts, the set of contigs corresponding to the assembled genome of the Puno-proglottid and the set of contigs corresponding to the hybrid assembly created from both the Huancayo and Puno tissue genome assemblies is available together at Genbank under the project ID PRJNA183343.

### Identification of microsatellites in the *T*. *solium* genome

Microsatellites were identified using the script developed by Gur-Arie *et al* [31]. We searched for repetitive motifs of 1–6 bp in our two assembled *T*. *solium* genomes. A total of 36 distinct microsatellites with polymorphic attributes were selected according to the following criteria: For the Puno-proglottid genome, a first group of microsatellites with a minimum of five motif repetitions were selected. In order to determine if the microsatellites are transcribed, we verified if they were present in the *T*. *solium* ESTs sequences database available (http://www.ncbi.nlm.nih.gov/nucest/?term=%22Taenia+solium%22%5Bporgn%3A__txid6204%5D), which later was included in the published *T*. *solium* genome sequence [28]. In order to determine if the microsatellites are comprised within a coding region, we verified the presence of an ORF using the algorithm ORF-Finder (http://www.ncbi.nlm.nih.gov/gorf/gorf.html). Microsatellites that showed differences in the number of repeats between the Puno-proglottid genome and the ESTs database were selected for further analysis. We verified that conserved flanking regions were present in both the Puno-proglottid genome and the ESTs database. Five microsatellites (TS_SSR01 to T S_SSR05) were selected. In addition, 8 of the longest microsatellite sequences from the Puno-proglottid genome not present in the ESTs database also were selected (TS_SSR06 to TS_SSR13). Because this first group of microsatellites was biased based on their availability in ESTs, it is less likely that they are neutral.

A second group of microsatellites was identified in the Puno-proglottid genome and mapped into the corresponding contigs of the Huancayo cyst genome. 200 microsatellites (100 di-nucleotides and 100 tri-nucleotides) with the largest repetitive motif in both genomes were evaluated. After aligning the sequences we selected the largest sequences that showed polymorphisms in the repetitive sequences between both genomes (13–18 repeats). This resulted in additional 23 microsatellite loci for testing.

#### Primer design for microsatellite amplification

In order to prevent any bias due to the assembly of a consensus genome that does not account for the intrinsic genomic variability due to the diplodism of *T*. *solium* and the potentially different sources of infection giving raise to the multiple cysticercus processed to obtain DNA, we directly determined the size of the microsatellites after the individual amplification in each sample. Specific primers were designed against the conserved flanking sequences of each microsatellite. The Primer3 program was used with the following parameters: 20 bp length, 50% GC and 50°C Tm [32].

#### Tapeworm specimens

A total of 40 *T*. *solium* adult tapeworm specimens were randomly and anonymously selected from the repository of the Cysticercosis Working Group in Peru. Isolates came from different communities from the northern city of Peru, Tumbes (N = 20) and the southern city of Peru, Puno (N = 20) (Table 1). The geographical distribution of the collected *T*. *solium* tapeworm isolates is shown in Fig 1. Tapeworms were stored in 25% glycerol supplemented with penicillin (1000 UI/mL) and Gentamycin (100 μg/mL) at 4°C until use.

**Table 1:** Origin of *T*. *solium* tapeworm isolates for the evaluation of microsatellites.

Tapeworm ID	Community	Populations †	Region
6	San Isidro	Cruz-Isidro-pinos	Tumbes
39	San Isidro	Cruz-Isidro-pinos	Tumbes
28	San Isidro	Cruz-Isidro-pinos	Tumbes
38	La Cruz	Cruz-Isidro-pinos	Tumbes
7	Los Pinos	Cruz-Isidro-Pinos	Tumbes
19	Tumbes	Tum-Corrales-San Juan-PenaBlanca	Tumbes
31	Tumbes	Tum_corrales-san juan_PenaBlanca	Tumbes
20	Corrales	Tum_corrales-san juan_PenaBlanca	Tumbes
27	Corrales	Tum_corrales-san juan_PenaBlanca	Tumbes
11	Corrales	Tum_corrales-san juan_PenaBlanca	Tumbes
12	Fuerte 5 de Julio	Tum_corrales-san juan_PenaBlanca	Tumbes
5	San Juan—Virgen	Tum_corrales-san juan_PenaBlanca	Tumbes
25	Peña Blanca	Tum_corrales-san juan_PenaBlanca	Tumbes
18	La Choza	Cañaveral–Choza	Tumbes
24	Cañaveral	Cañaveral–Choza	Tumbes
14	Pueblo Nuevo	Uñagato-progreso-pueblonuevo	Tumbes
36	Pueblo Nuevo	Uñagato-progreso-pueblonuevo	Tumbes
8	Nuevo Progreso	Uñagato-progreso-pueblonuevo	Tumbes
35	Nuevo Progreso	Uñagato-progreso-pueblonuevo	Tumbes
13	Uña de Gato	Uñagato-progreso-pueblonuevo	Tumbes
40	Pharata	Fharata	Puno
1	Pharata	Fharata	Puno
30	Pharata	Fharata	Puno
17	Pharata	Fharata	Puno
21	Pharata	Fharata	Puno
34	Pharata	Fharata	Puno
3	Callata	Callata	Puno
9	Callata	Callata	Puno
10	Callata	Callata	Puno
29	Callata	Callata	Puno
4	Callata	Callata	Puno
15	Camicachi	Camicachi	Puno
33	Camicachi	Camicachi	Puno
37	Camicachi	Camicachi	Puno
23	Camicachi	Camicachi	Puno
32	Camicachi	Camicachi	Puno
16	Conchaca	Conchaca_Tuturuma	Puno
2	Conchaca	Conchaca_Tuturuma	Puno
26	Conchaca	Conchaca_Tuturuma	Puno
22	Tuturuma	Conchaca_Tuturuma	Puno

† Populations are the groups that were formed by proximity for the phylogenetic analysis

#### DNA purification and PCR amplification of microsatellite loci

DNA purification was performed using the QIAmp DNA Mini Kit (QIAGEN) according to manufacturer´s instructions. DNA was quantified by UV spectrophotometry (Nanodrop) and stored at -20°C until use. The PCR reaction volume (25 μL) consisted of: Buffer 1X (Promega), 2.5 mM MgCl_2_, 0.2 mM dNTPs each one, Forward primer: 1μmol, Reverse primer: 1 μmol and 20 ng of DNA, Taq polymerase 1 U. PCR was conducted in a MJ Research MiniCycler PTC-150 thermocycler with a hot cover using the following temperature profile except where otherwise stated: the initial denaturation step was at 94°C for 4 minutes, followed by 30 cycles of 94°C for 1 minute, 55–65°C for 1 minute and 72°C for 1 minute and a final extension at 72°C for 10 minutes. Microsatellite markers were analyzed in a multi-capillary electrophoresis QIAxcel system using the QIAxcel high-resolution kit with the OM700 method [29]. 25 bp-500 bp ladder was used as a size marker for the assignment of the allele sizes using the system’s software. As a control and in order to verify reproducibility, we amplified the microsatellite TS_SSR01 in four independent replicates from 9 DNA samples.

#### Association analysis of microsatellite polymorphism with geographic location

In order to determine an association between the geographic origins of the tapeworms with the specific microsatellites, we performed a Fischer’s exact test to evaluate the independence between the alleles identified in each microsatellite and the north/south geographic origin.

### Ethics statement

*Taenia solium* cysts were excided in a previous study from a naturally infected pig in a Huancayo local abattoir. The pig was bought by the study team at market price so the study team owned the animal. Procedures were approved by Universidad Peruana Cayetano Heredia (UPCH) ethics committee for animal use *T*. *solium* proglottid specimens were collected in previous studies by the Cysticercosis Working Group in Peru, with approval of the UPCH IRB (IRB00001014); they were used as residual diagnostic samples.

## Results

### Analysis of genomic assemblies

Sequencing of the Huancayo-cyst genome produced a total of 175,931,369 reads that were assembled into 18,361 contigs (the largest contig was 307,365 bp). The estimated genome size was 114,605,177 nt. The sequencing of the Puno-proglottid genome produced a total of 76,625,473 reads that were assembled in 47,475 contigs (the largest contig was 79,438 nt). The lack of large contigs in this assembly is due to the lack of paired-end sequencing data. The estimated size of the Puno-proglottid genome was 109,898,809 nt. For the hybrid cyst/proglottid assembly, 7,979 contigs were obtained, and the largest contig had 395,362 nt. The estimated size of the hybrid genome was 111,029,218 nt. These and other statistics were calculated with the program multifastats,py v1.4 (https://github.com/lbbm-upch/multifastats_v1.4), and are summarized in Table 2. As expected due to their closeness, the Peruvian *T*. *solium* genomes showed a similar size as the recently published Mexican *T*. *solium* genome (122.3 Mb), as well as the genomes of *E*. *granulosus* (114.9 Mb), and *E*. *multilocularis* (115 Mb) [28].

**Table 2:** *Taenia solium* genomes assemblies’ statistics.

	CDC	NIH	Hybrid	Hybrid
	Puno proglottid	Huancayo Cyst	Cyst/proglottid (all contigs)	Cyst/proglottid (GenBank)†
Total sequenced bases	109,898,809	114,605,177	116,668,703	111,029,218
Contigs	47,475	18,361	19,727	7,979
GC%	42.96	42.83	42.86	42.80
N50	4,839	39,744	46,836	43,923
Shortest contig (nt)	287	51	51	1000
Largest contig (nt)	79,438	307,365	395,362	39,5362
Mean contig length	2,314.9	6,241.8	5,914.2	13,915.2

Statistics of the hybrid genome are shown for the full version and for the set of contigs uploaded to GenBank

†Only contigs larger than 1000bp were submitted

### Identification of microsatellites

We identified 9,129 microsatellite sequences distributed in the *T*. *solium* Puno-proglottid genome and 9,936 in the Huancayo-cyst Genome (Table 3). In both the Puno and Huancayo genomes, the greatest number of microsatellite loci found contained di-nucleotides repeats. Most of these microsatellites were over-represented in the forms of AC/GT and AG/CT, while the forms AT/AT and CG/CG showed a lower frequency of occurrence (S1 Table).

**Table 3:** Frequency of microsatellites per type found in *T*. *solium* partial Genome 1 and partial Genome 2.

Number of nucleotides	Range of repeats	Number of microsatellites	
		Genome 1	Genome 2
Total bp analized		109’898,809	114,605,177
Mono nucleotide	10 to 53	2,298	2,737
Di nucleotide	6 to 38	3,393	3,537
Tri nucleotide	5 to 28	2,393	3,464
Tetra nucleotide	5 to 28	889	1,044
Penta nucleotide	5 to 18	123	122
Hexa nucleotide	5 to 10	33	32
Total		9,129	9,936

### Microsatellite PCR and polymorphism analysis

Thirty-six microsatellites markers were identified as potentially polymorphic. We successfully amplified 34 microsatellite markers in 40 *T*. *solium* tapeworm specimens. The estimated size of the PCR products of the microsatellites in the tapeworm isolates was similar to the expected theoretical sizes.

Within the tested sample, twenty-seven microsatellite markers were monomorphic, of which 26 were homozygous (only one band was observed in the electrophoretic pattern) and 1 was heterozygous (TS_SSR31, in which two bands were observed in all samples). Seven microsatellite markers were polymorphic containing a total of 44 alleles within the polymorphic loci (Table 4). All 40 tapeworms were homozygous for five markers (TS_SSR09, TS_SSR16, TS_SSR18, TS_SSR27, and TS_SSR28), while some were heterozygous for TS_SSR01 and TS_SSR32 (S2 Table). The number of alleles varied from 4 for locus TS_SSR16 to 10 for locus TS_SSR32 with an average of 6 alleles per locus. The polymorphic information content (PIC) varied from 0.472 for locus TS_SSR16 to 0.843 for locus TS_SSR28 with an average of 0.604 per locus (Table 4).

**Table 4:** Characteristics of microsatellite loci evaluated.

Microsatellite	Primer sequences (5'-3')	Repetition motif	Observed size (bp)	Number of Alleles	PIC ±
TS_SSR_01	ACCGGTGGTCGGAATTATTA GTTCTTGCTGAGGTGGTTCC	(CCATT)	206–226	5	0.582
TS_SSR_02	CTCCGTGTCTTGACAGCAAA TGACGAAATGGAACAGTGGA	(ATGA)	190	1	-
TS_SSR_03	TTTCAAGCACGTGTCAGCAT GCTGGCAGACAGTGAGTAGG	(CATT)	155	1	-
TS_SSR_04	CAGATGAGGGGATGATGCTT GAACGATCCCAACCTCCATA	(GTT)	180	1	-
TS_SSR_05	GGGAAAATGCAGTTCAGAGC GGTCTGATGCGAGGTCTAGG	(TAA)	197	1	-
TS_SSR_06	GACCAAGCCCAACACCTCTA CAAGAATGAACGGGAGCAAC	(GGTA)	177	1	-
TS_SSR_07	GCACACAAACTGGTCACTCG TGCTATGCGTTTGCTTGTTT	(CAAT)	--	-	-
TS_SSR_08	TCGTCAGTGTGGGAGAGTGA TGGTTGGATTTGTGCTTTGA	(ACG)	--	-	-
TS_SSR_09	AAGCCAATGGTGACCAAGAG GCCAGCATAGAAGAGCCTGT	(GGT)	166–178	5	0.534
TS_SSR_10	CGACTCACGGCATTCATCTA TCCAAGACCCTGTGAAATCC	(GT)	220	1	-
TS_SSR_11	TCATCTTCCCCGTAAGGCTA ACACTCGAAGCGCAGTGTTT	(GA)	181	1	-
TS_SSR_12	ATCTCGACAGGCTCGAGTTC TCCGAACAGCTTCGAGTTTT	(TG)	192	1	-
TS_SSR_13	GTAGCGGTAACGGAGTGAGG TCAGGCTGGTAACGTGTCAG	(GT)	202	1	-
TS_SSR_14	AGCCGGTTCTCAGTTGATTG AATGCACTCATGCCATCTCA	(TG)	162	1	-
TS_SSR_15	GAAAAGAACGGCATGCAAAT GTTTGGCCATTTTGCCTCTA	(AT)	165	1	-
TS_SSR_16	CGCTGGACTAGGGTCGAATA CAGCAGAACAACAGCACCAT	(GT)	160–166	4	0.472
TS_SSR_17	GCATTCCGAGGATGAATGAT CGTTTTTCTGCACACTTGGA	(CA)	160	1	-
TS_SSR_18	AGTTAGCGTGCTTGCTTGGT ATTCCTGTTGCAACCTCCAC	(GT)	168–180	6	0.638
TS_SSR_19	TCCCTTACACCCTTCACGTC AAAGGCGGTAGATTGTGTGC	(TG)	163	1	-
TS_SSR_20	GGCCATTCAGTACCAACCAT TGTGCATGCCATTGTATGTG	(CT)	154	1	-
TS_SSR_21	CTATGCCACACCCAACAATG GGCCTTCAAGATCACTCGTC	(GT)	187	1	-
TS_SSR_22	CCTATTCCACTGGGGTGATG TCGATGAGCTTGCTGTATGTG	(TG)	178	1	-
TS_SSR_23	CCTTTTTCGGTGAAGTCGAT GCCTCCTTACACACATGCAA	(CA)	209	1	-
TS_SSR_24	CCCCATTTCCTGTTTCCTCT GCGGTGGCAATATAAGCATT	(CT)	144	1	-
TS_SSR_25	AGGTGGCGTTATGAATCAGC GCAAACCATCGGATAAAGGA	(AC)	174	1	-
TS_SSR_26	CGGTTTGCTTTTATGCCAAT AAATGGTCGCCTGAAATGAC	(GAA)	165	1	-
TS_SSR_27	GAGGTCTCGCCTCATCAAAG TTTCCACTCCCAAAAACTCG	(GAA)	158–176	5	0.546
TS_SSR_28	TGACGCTGGTAAGCTGTTTG GGAACCTTGGCACGAGATAG	(GTA)	202–226	9	0.843
TS_SSR_29	AAAGATGGACGGAAACAGGA GTTGGACGGAGATGTGTGTG	(AGG)	187	1	-
TS_SSR_30	TGACGTGTCGTCAGGTAGGA CGCATAGCCAGTACTTGTTCC	(TCC)	190	1	-
TS_SSR_31	GGTTGCTTTTGCTTGTCCTC CACTCTCCACGAGTCCACAA	(TGA)	157/179	1	-
TS_SSR_32	TGACGTTAACGAGGGTGTTG AGATCTCGCCTTGCAACAAT	(AGC)	177–210	10	0.617
TS_SSR_33	CCAGCGGCATATTACAAAGG ACTCAAAAGCGCCGAAATTA	(AGG)	130	1	-
TS_SSR_34	ATCACTCCTGTCCCAACTGC GGGTCGATTGGTCAGAGAAA	(CCT)	182	1	-
TS_SSR_35	GGGCGTGAACTCGAATAAAA GGGGCAGACAAGTGAAAAAG	(CCA)	170	1	-
TS_SSR_36	GCCCTGATTGTTGCTTTTGT AACGACACGCGGAAAATATC	(TCT)	175	1	-

± PIC was calculated only for polymorphic microsatellites

The reproducibility analysis of TS_SSR01 amplification showed a variability of 1–2 bp between the four replicas in the nine isolates tested (S3 Table), which is lower than the range of resolution reported by the manufacturer (3–5 bp). The variability of TS_SSR01 size between the different *T*. *solium* isolates appeared as 2–15 bp, which is three fold higher than the experimental error reported by the manufacturer (S3 Table).

Only TS_SSR01 was found to be present in the EST database. However the comprising region did not show evidence of any ORF. Therefore TS_SSR01 is part of a transcribed but not-translated sequence. When we compared TS_SSR01 against the complete no-redundant nucleotide collection of Genbank using blastn, only one sequence from the close organism *T*. *asiatica* appeared similar.

### Association of microsatellite polymorphism with the geographic origin of tapeworms

The genetic diversity observed in isolates from the southern city of Puno (20 different genotypes) was slightly higher than that the genetic diversity observed in the isolates from the northern city Tumbes (16 different genotypes). Also the median number of alleles was slightly higher (Table 5). The single microsatellite that best differentiated tapeworms from Tumbes and Puno isolates was TS_SSR01. Most of the isolates from Tumbes (17/20) were associated to genotype A (206/206 bp) and 3/20 of isolates were associated to genotype B (211/211 bp), all of them homozygous. A lower prevalence of genotype A was observed in Puno (6/20) compared to Tumbes (P = 0.001, Fisher’s exact test). A similar prevalence of genotype B (2/20) was observed in Puno. Five other genotypes were found only in Puno (S2 Table).

**Table 5:** General characteristics of polymorphic microsatellites by region.

Region	Number of isolates	Number of polymorphic loci	Median number of alleles per locus	Total number of different genotypes
Tumbes	20	7	3	16
Puno	20	7	5	20

## Discussion

The present study describes the draft genome sequences of two *T*. *solium* isolates and the identification and characterization of DNA microsatellites. The *T*. *solium* microsatellites reported here were found to be distributed along the entire genome. The length polymorphism of microsatellites was analyzed for its association with the geographic origin of tapeworm isolates. We found novel microsatellites that were able to differentiate tapeworms between the northern and southern regions of Peru.

Microsatellites have proven to be highly informative in population genetic studies in several parasites [6,33]. In the particular case of *T*. *solium*, the use of microsatellite markers allows a way to define the genetic structure of populations and to conduct genetic epidemiology studies. Although previous studies have shown a moderate genetic diversity of *T*. *solium* [4,9,10,34] and particularly in Peru [12], the novel microsatellites we identified here have demonstrated the capacity to differentiate tapeworms from Tumbes in the north and Puno in the south of Peru.

Although the frequency of microsatellites and their coverage in the genome varies considerably between organisms, the number of microsatellites found in *T*. *solium* (between 9,000–10,000) is similar to the number of microsatellites identified in other parasites [21,35].

Although most of the microsatellite sequences were found in non-transcribed regions, we found that *T*. *solium* microsatellites could also be present in transcribed/non-translated regions, being the abundance in non-transcribing regions higher than in transcribed/non-translated regions. This result is consistent with previous studies that reported that microsatellites are more abundant in non-coding regions of eukaryotic organisms [36]. The relatively low abundance of microsatellites in transcribed/non-translated as well as in coding regions could be explained by a negative selection against mutations that change the function by altering the secondary structure of the transcribed sequence or by altering the reading frame of the genes [36,37].

Eukaryote microsatellite loci typically contain between 5 and 40 repeats, similar to what we found in *T*. *solium*. As in other organisms, the number of microsatellites in *T*. *solium* decreases as the size of the repeat unit increases [38,39]. It is important to highlight that the distribution of the repeat types (mono- to hexa-nucleotide) varies across different taxa, and it has been suggested that this variation is associated with to the interaction of the mutation and the differential selection pressure [37].

As previously reported in other species, we found that dinucleotide repeats motifs were the most abundant type in *T*. *solium*, which tend to be longer in non-coding regions. This seems to be explained by the negative selection pressure of polymerase slippage during replication of coding DNA [40]. Castagnone—Sereno *et al*. reported that in nematodes, (AT)n was the most common microsatellite motif [35]. We found the AC / GT dinucleotide motif to be the most abundant in *T*. *solium*, which concordantly has also been found to be common in most vertebrates and arthropods [41].

The genetic variability observed in this study may be explained by several factors, including migration of humans and pigs, mutations in the tapeworm genome, cross-fertilization of tapeworms in the intestine in cases where multiple tapeworm infections occur [42,43], among others.

It is important to note that although the low resolution of QIAxcel system reported by the company (3–5bp), the range of difference in the size of TS SSR01 between the north and south region of Peru, is 2–3 fold higher than the expected error (5–15 bp) and the results of the repeatability assay showed lower variability (1–2 bp). This evidence supports the main finding of having TS SSR01 as a polymorphic marker able to differentiate tapeworms from the north and south region of Peru.

Transmission dynamics are not fully understood, although genetic characterization by means of microsatellite genotyping may unveil details of the ecology of *T*. *solium*. The use of molecular characterization by means of microsatellites will potentially allow identification of genetic links between tapeworms, larval cysts found in infected pigs and eggs in soil or fomites. Furthermore, microsatellites would help disentangle the genetic complexity of a population due to the introduction of external tapeworms from immigrant tapeworm-carriers. This method of genotyping also has implications in the evaluation of parasite control by identifying the source of infection, and the re-introduction routes of the parasite into a specific region.

In conclusion, this study describes the identification and application of microsatellite markers in *T*. *solium* genotyping. The novel microsatellites reported here would be an important tool for future studies of the genetic variability of *T*. *solium*, including population genetics, basic epidemiology, super infections with more than one strain, and tracking the transmission of cysticercosis.

## Supporting Information

S1 Table Relative frequency of different motifs in each type of microsatellites: mono-, di- and tri- nucleotides in the partial genomes of *Taenia solium*. (DOCX)

S2 Table Genotype of the 40 *Taenia solium* isolates for each polymorphic microsatellite marker. (DOCX)

S3 Table Repeatability assay of microsatellite TS_SSR01. (DOCX)
