# Supplementary material for: Identification and Characterization of Microsatellite Markers Derived from the Whole Genome Analysis of Taenia solium
Source: PLoS Negl Trop Dis. 2015 Dec 23;9(12):e0004316. doi: 10.1371/journal.pntd.0004316 (PMC4689449; doi:10.1371/journal.pntd.0004316)
Supplement: S1 Table — (DOCX) [file pntd.0004316.s001.docx]

**Table S1.** Relative frequency of different motifs in each type of microsatellites: mono-, di- and tri- nucleotides in the partial genomes of *Taenia solium*

| **Type of repetition** | **Genome 1** | **Genome 2** |
| --- | --- | --- |
| **Mono-nucleotide** |  |  |
| A/T | 0,85 | 0,78 |
| C/G | 0,15 | 0,22 |
| **Di-nucleotide** |  |  |
| AC/GT | 0,69 | 0,67 |
| AG/CT | 0,24 | 0,26 |
| AT/AT | 0,07 | 0,07 |
| CG/CG | 0,00 | 0,00 |
| **Tri-nucleotide** |  |  |
| AAC/GTT | 0,09 | 0,09 |
| AAG/CTT | 0,11 | 0,11 |
| AAT/ATT | 0,05 | 0,05 |
| ACC/GGT | 0,18 | 0,19 |
| ACG/CGT | 0,02 | 0,02 |
| ACT/AGT | 0,03 | 0,03 |
| AGC/CTG | 0,10 | 0,08 |
| AGG/CCT | 0,34 | 0,35 |
| ATC/ATG | 0,06 | 0,06 |
| CCG/CGG | 0,02 | 0,01 |
