# Supplementary material for: Identification and Characterization of Microsatellite Markers Derived from the Whole Genome Analysis of Taenia solium
Source: PLoS Negl Trop Dis. 2015 Dec 23;9(12):e0004316. doi: 10.1371/journal.pntd.0004316 (PMC4689449; doi:10.1371/journal.pntd.0004316)
Supplement: S2 Table — (DOCX) [file pntd.0004316.s002.docx]

**Table S2. Genotype of the 40 *Taenia solium* isolates for each polymorphic microsatellite marker**

| **Code** | **Community** | **Department** | **TS_SSR01** | | **TS_SSR09** | | **TS_SSR16** | | **TS_SSR18** | | **TS_SSR27** | | **TS_SSR28** | | **TS_SSR32** | |
| --- | --- | --- | --- | --- | --- | --- | --- | --- | --- | --- | --- | --- | --- | --- | --- | --- |
| 6 | San Isidro | Tumbes | 206 / | 206 | 166 / | 166 | 160 / | 160 | 168 / | 168 | 161 / | 161 | 217 / | 217 | 180 / | 180 |
| 39 | San Isidro | Tumbes | 211 / | 211 | 166 / | 166 | 160 / | 160 | 168 / | 168 | 161 / | 161 | 220 / | 220 | 180 / | 180 |
| 28 | San Isidro | Tumbes | 206 / | 206 | 166 / | 166 | 160 / | 160 | 168 / | 168 | 161 / | 161 | 220 / | 220 | 180 / | 180 |
| 38 | La Cruz | Tumbes | 206 / | 206 | 166 / | 166 | 160 / | 160 | 168 / | 168 | 158 / | 158 | 220 / | 220 | 180 / | 180 |
| 7 | Los Pinos | Tumbes | 206 / | 206 | 166 / | 166 | 160 / | 160 | 168 / | 168 | 158 / | 158 | 214 / | 214 | 180 / | 180 |
| 19 | Tumbes | Tumbes | 206 / | 206 | 166 / | 166 | 160 / | 160 | 168 / | 168 | 161 / | 161 | 217 / | 217 | 180 / | 180 |
| 31 | Tumbes | Tumbes | 206 / | 206 | 166 / | 166 | 160 / | 160 | 168 / | 168 | 161 / | 161 | 223 / | 223 | 177 / | 192 |
| 20 | Corrales | Tumbes | 206 / | 206 | 166 / | 166 | 160 / | 160 | 168 / | 168 | 164 / | 164 | 214 / | 214 | 180 / | 180 |
| 27 | Corrales | Tumbes | 206 / | 206 | 166 / | 166 | 160 / | 160 | 168 / | 168 | 158 / | 158 | 220 / | 220 | 180 / | 180 |
| 11 | Corrales | Tumbes | 206 / | 206 | 169 / | 169 | 162 / | 162 | 168 / | 168 | 161 / | 161 | 220 / | 220 | 183 / | 183 |
| 12 | Corrales | Tumbes | 206 / | 206 | 169 / | 169 | 164 / | 164 | 170 / | 170 | 161 / | 161 | 220 / | 220 | 180 / | 180 |
| 5 | San Juan | Tumbes | 206 / | 206 | 166 / | 166 | 160 / | 160 | 168 / | 168 | 158 / | 158 | 214 / | 214 | 180 / | 180 |
| 25 | Peña Blanca | Tumbes | 206 / | 206 | 169 / | 169 | 160 / | 160 | 170 / | 170 | 161 / | 161 | 220 / | 220 | 183 / | 183 |
| 18 | La Choza-Casitas | Tumbes | 211 / | 211 | 166 / | 166 | 160 / | 160 | 172 / | 172 | 161 / | 161 | 217 / | 217 | 180 / | 180 |
| 24 | Cañaveral | Tumbes | 211 / | 211 | 169 / | 169 | 162 / | 162 | 170 / | 170 | 161 / | 161 | 211 / | 211 | 183 / | 183 |
| 14 | Pueblo Nuevo | Tumbes | 206 / | 206 | 166 / | 166 | 160 / | 160 | 172 / | 172 | 158 / | 158 | 223 / | 223 | 180 / | 180 |
| 36 | Pueblo Nuevo | Tumbes | 206 / | 206 | 169 / | 169 | 162 / | 162 | 170 / | 170 | 164 / | 164 | 223 / | 223 | 183 / | 183 |
| 8 | Nuevo Progreso | Tumbes | 206 / | 206 | 166 / | 166 | 160 / | 160 | 168 / | 168 | 158 / | 158 | 214 / | 214 | 180 / | 180 |
| 35 | Nuevo Progreso | Tumbes | 206 / | 206 | 169 / | 169 | 162 / | 162 | 168 / | 168 | 161 / | 161 | 217 / | 217 | 183 / | 183 |
| 13 | Uña de gato-Papayal | Tumbes | 206 / | 206 | 169 / | 169 | 160 / | 160 | 180 / | 180 | 158 / | 158 | 223 / | 223 | 183 / | 183 |
| 40 | Pharata | Puno | 216 / | 226 | 166 / | 166 | 160 / | 160 | 170 / | 170 | 161 / | 161 | 208 / | 208 | 180 / | 180 |
| 1 | Pharata | Puno | 211 / | 226 | 178 / | 178 | 166 / | 166 | 176 / | 176 | 173 / | 173 | 202 / | 202 | 186 / | 210 |
| 30 | Pharata | Puno | 211 / | 211 | 166 / | 166 | 160 / | 160 | 172 / | 172 | 161 / | 161 | 205 / | 205 | 180 / | 204 |
| 17 | Pharata | Puno | 206 / | 206 | 175 / | 175 | 160 / | 160 | 172 / | 172 | 161 / | 161 | 202 / | 202 | 192 / | 207 |
| 21 | Pharata | Puno | 206 / | 206 | 172 / | 172 | 160 / | 160 | 172 / | 172 | 161 / | 161 | 205 / | 205 | 180 / | 195 |
| 34 | Pharata | Puno | 206 / | 206 | 175 / | 175 | 160 / | 160 | 172 / | 172 | 161 / | 161 | 217 / | 217 | 183 / | 207 |
| 3 | Callata | Puno | 226 / | 226 | 178 / | 178 | 164 / | 164 | 172 / | 172 | 173 / | 173 | 205 / | 205 | 189 / | 207 |
| 9 | Callata | Puno | 216 / | 221 | 166 / | 166 | 164 / | 164 | 172 / | 172 | 161 / | 161 | 226 / | 226 | 180 / | 180 |
| 10 | Callata | Puno | 211 / | 221 | 166 / | 166 | 164 / | 164 | 172 / | 172 | 161 / | 161 | 226 / | 226 | 183 / | 183 |
| 29 | Callata | Puno | 211 / | 211 | 166 / | 166 | 160 / | 160 | 172 / | 172 | 161 / | 161 | 205 / | 205 | 180 / | 207 |
| 4 | Callata | Puno | 206 / | 206 | 166 / | 166 | 164 / | 164 | 176 / | 176 | 176 / | 176 | 205 / | 205 | 180 / | 204 |
| 15 | Camicachi | Puno | 211 / | 221 | 166 / | 166 | 160 / | 160 | 172 / | 172 | 161 / | 161 | 208 / | 208 | 180 / | 207 |
| 33 | Camicachi | Puno | 216 / | 226 | 166 / | 166 | 160 / | 160 | 172 / | 172 | 161 / | 161 | 202 / | 202 | 180 / | 204 |
| 37 | Camicachi | Puno | 216 / | 221 | 172 / | 172 | 160 / | 160 | 176 / | 176 | 161 / | 161 | 220 / | 220 | 183 / | 183 |
| 23 | Camicachi | Puno | 226 / | 226 | 169 / | 169 | 162 / | 162 | 172 / | 172 | 158 / | 158 | 205 / | 205 | 183 / | 183 |
| 32 | Camicachi | Puno | 206 / | 206 | 166 / | 166 | 164 / | 164 | 172 / | 172 | 176 / | 176 | 205 / | 205 | 180 / | 204 |
| 16 | Conchaca | Puno | 211 / | 221 | 166 / | 166 | 164 / | 164 | 172 / | 172 | 161 / | 161 | 226 / | 226 | 180 / | 180 |
| 2 | Conchaca | Puno | 216 / | 221 | 169 / | 169 | 160 / | 160 | 172 / | 172 | 164 / | 164 | 202 / | 202 | 189 / | 207 |
| 26 | Conchaca | Puno | 206 / | 206 | 166 / | 166 | 164 / | 164 | 174 / | 174 | 176 / | 176 | 205 / | 205 | 180 / | 207 |
| 22 | Tuturuma | Puno | 216 / | 226 | 175 / | 175 | 160 / | 160 | 172 / | 172 | 161 / | 161 | 217 / | 217 | 180 / | 180 |

Genotypes are presented with the size bands of the PCR products for each isolate
