# Supplementary material for: Identification and Characterization of Microsatellite Markers Derived from the Whole Genome Analysis of Taenia solium
Source: PLoS Negl Trop Dis. 2015 Dec 23;9(12):e0004316. doi: 10.1371/journal.pntd.0004316 (PMC4689449; doi:10.1371/journal.pntd.0004316)
Supplement: S3 Table — (DOCX) [file pntd.0004316.s003.docx]

**Table S3.** Reproducibility assay of microsatellite TS_SSR01

| **Sample** | **Repetition** | | | |
| --- | --- | --- | --- | --- |
|  | **1** | **2** | **3** | **4** |
| 2 | 216/221 | 216/221 | 216/221 | 215/220 |
| 3 | 228 | 227 | 227 | -† |
| 4 | 206 | 206 | 206 | 207 |
| 5 | 206 | 206 | 206 | -† |
| 6 | 206 | 206 | 206 | 206 |
| 7 | 206 | 206 | 206 | 206 |
| 8 | 206 | 206 | 206 | 206 |
| 9 | 216/221 | 216/220 | 216/220 | 217/221 |
| 10 | 211 /221 | 211/221 | 211/221 | 211/221 |

Bands sizes were obtained with 4 different PCR using TS_SSR01 in samples 2 to 10. Electrophoresis was performed on the QIAxcel System.

† Samples did not amplify.
